# OA24 Beyond Remission: A Multidisciplinary Approach to Transition Care in Juvenile Idiopathic Arthritis

**DOI:** 10.1093/rap/rkag113.024

**Published:** 2026-11-03

**Authors:** Chethana Ramakrishna, Laura Flower, Jacqui Tomkins, Alice Mason

**Affiliations:** University Hospital Southampton, Southampton, United Kingdom; University Hospital Southampton, Southampton, United Kingdom; University Hospital Southampton, Southampton, United Kingdom; University Hospital Southampton, Southampton, United Kingdom

## Abstract

**Introduction:**

The transfer of adolescents with chronic rheumatic conditions, most commonly Juvenile Idiopathic Arthritis (JIA) from paediatric to adult healthcare requires careful planning to ensure continuity of care and promote long-term health outcomes. While disease control is an important objective, successful transition also depends on addressing psychological well-being, education, physical function and the development of self-management skills. Young people often encounter challenges adapting to adult services, highlighting the need for coordinated multidisciplinary support. Dedicated transition and young adult clinics play an important role in delivering holistic, patient-centred care that extends beyond medical management.

**Case description:**

A young adult woman (aged 23 years) with polyarticular JIA, diagnosed at 11 years of age, attended a multidisciplinary young adult clinic, as part of the ongoing transfer process from paediatric to adult rheumatology services. Throughout her disease course, she received several immunosuppressive therapies, including methotrexate, etanercept, adalimumab, and infliximab, before achieving sustained remission with oral baricitinib, which remains her current treatment. At review, clinical examination demonstrated no active synovitis, she reported no recent disease flares, and routine laboratory investigations were within normal limits, indicating stable disease and well-controlled inflammatory arthritis.

Despite excellent disease control, the consultation highlighted broader concerns that were of greater importance to the patient than her arthritis. She described difficulties obtaining employment and uncertainty regarding future career options, reflecting common challenges experienced during transition to adulthood. The multidisciplinary team, including a clinical psychologist, provided guidance on employment support services, volunteering opportunities, and youth organisations.

The patient also reported reduced muscle strength and poor physical fitness, affecting her confidence and daily functioning. Following physical assessment, the therapy team recommended an individually tailored programme of graded exercise to improve strength, physical conditioning, and confidence in physical activity.

The rheumatology consultant addressed other important care aspects, such as contraception and immunisation, ensuring comprehensive management beyond disease control. The clinic incorporates a structured transition questionnaire to evaluate patients’ understanding of their condition, confidence in self-management, readiness for adult services, and priorities for discussion. Standardised screening tools, including the GAD-7 and PHQ-8 questionnaires, are completed before appointments to identify anxiety, depression, and other psychological concerns that may otherwise remain unrecognised. This multidisciplinary, structured approach supports personalised, holistic care by addressing the medical, psychological, and social needs of young people during the transition from paediatric to adult rheumatology services.

**Discussion:**

The transition from paediatric to adult rheumatology care represents a critical period for young people with chronic rheumatic diseases. Although advances in treatment have significantly improved disease outcomes, achieving optimal long-term health requires more than effective suppression of inflammation. Adolescence and early adulthood involve major developmental changes, including establishing independence, pursuing education or employment, maintaining physical health, and developing confidence in self-management. These factors can have a considerable impact on quality of life and should be considered integral components of transition care.

This case highlights that even with excellent disease control and sustained remission, young adults with JIA may experience significant challenges related to their condition. This patient’s concerns regarding employment, future career opportunities, and reduced physical fitness were unrelated to inflammatory disease activity but represented important barriers to wellbeing and successful adaptation to adult life.

Multidisciplinary transition clinics provide an opportunity to address the complex needs of young people. Collaboration between rheumatologists, psychologists, and physiotherapists enables care to be individualised and focused on patient priorities. In this case, psychological support facilitated access to employment resources and youth support networks, while physiotherapy input enabled a graded exercise programme to improve strength, confidence, and functional ability. Structured transition assessments and validated psychological screening tools, including the GAD-7 and PHQ-8, can identify unmet needs that may not emerge during routine consultations and support early intervention.

Overall, this case demonstrates that successful transition is not simply the transfer of medical care from paediatric to adult services, but a process of empowering young people to manage their condition while addressing the wider factors influencing health, independence, and quality of life. This multidisciplinary approach aligns with a recently published British Society for Rheumatology guideline scope for the holistic management of JIA across all ages, which emphasises comprehensive, patient-centred care extending beyond pharmacological disease control.

**Key learning points:**

• Successful transition is not simply the transfer of medical care from paediatric to adult services, but a process of empowering young people to manage their condition, while addressing wider factors which can influence health.

• Transition from paediatric to adult rheumatology care occurs at a developmentally critical period. Young people with chronic, rheumatic conditions need to learn to manage their physical health conditions during this often turbulent time.

• Patient concerns frequently extend beyond the direct impact of their disease to potential indirect effects including education and employment, physical fitness, psychological well-being, personal relationships and concerns for the future.

• Using the multidisciplinary model in the outpatient clinic setting can ensure that the right clinician is seeing the patient to meet their needs at that time.

• Structured transition assessments and validated psychological screening tools can help ensure the consultation is patient centred and highlight issues that may otherwise have been missed.

